# Disparities in Early Childhood Caries

**DOI:** 10.1186/1472-6831-6-S1-S3

**Published:** 2006-06-15

**Authors:** Clemencia M Vargas, Cynthia R Ronzio

**Affiliations:** 1 Department of Health Promotion and Policy, University of Maryland Dental School, Baltimore, MD 21201, USA; 2 Center for Health Services and Community Research, Children's National Medical Center/George Washington University Medical Center, Washington, DC 20010, USA

## Abstract

Despite remarkable reduction in the prevalence of dental caries in the United States, dental caries is still a highly prevalent disease among children who are socially disadvantaged (racial/ethnic minority, poor, rural, immigrants). Consequently, caries sequelae such as dental pain, need for dental treatment under general anesthesia, and future orthodontic treatment, are also concentrated among the most socially disadvantaged children. To make the situation more appalling, those children who need treatment the most are the ones least likely to visit the dentist. Low income children are less likely to visit the dentist in part because of family's competing needs for limited resources, shortage of pediatric dentists, and dentists not taking uninsured or publicly insured patients. In the same vein, if these children do not have access to dental care, they are deprived from effective caries preventive measures that are dentist-dependent such as sealants and professionally applied fluoride. Dentistry has done well at devising caries preventive and treatment strategies; but these strategies have missed the most needed segment of society: disadvantaged children. The challenge now is to develop innovative strategies to reach these children.

## Introduction

Disparity in young children's oral health tells a story of the most vulnerable of the vulnerable. As a group, young children constitute a vulnerable population because of their dependence, their inability to communicate needs, and their relative poverty [1]. Although disparity amongst children can be found in nearly every marker of health, disparity in oral health is particularly disturbing because dental caries is the most common chronic disease of childhood [2].

What do we mean by disparity? Health disparity exists when one group receives a disproportionate share of a health burden. Disparities can be most commonly found along socioeconomic and race/ethnic lines. How this reveals itself in pediatric oral health, in access to dental care, and in policy and research implications is the focus of this review.

### Epidemiology

Because Early Childhood Caries (ECC) is, for the most part, a preventable disease, one could make the case that any incidence of ECC is a failure of the oral health system. But even within the context of unmet need, there are important disparities that deserve closer attention. In this section we describe the prevalence of ECC and treatment issues relevant to oral health disparity.

Different names and definitions have been used to refer to the presence of dental caries among very young children [3]. Currently, ECC is defined as "the presence of one or more decayed (cavitated or not cavitated lesions), missing (due to caries), or filled tooth surfaces in any primary tooth in a child 71 months of age or younger" [4]. In the United States, 23.7% of children 2–5 years of age have had caries experience [5], and 18.7% have untreated decay [6]. It is important to note that the prevalence of ECC reported by most U.S. studies does not include non-cavitated lesions or white spot lesions. Therefore, it is expected that the true prevalence of ECC is well above published estimates.

ECC is not evenly distributed across the U.S. population [7]. Regardless of the ECC definition used, there is overwhelming evidence that ECC is more prevalent among low-income and minority children. The third National Health and Nutrition Examination Survey (NHANES III) (conducted during 1988–94) showed that, in the U.S., the percentage of all children 2 to 5 years of age with untreated decay declines consistently as the family income increases. Thirty percent of children below the poverty line have untreated decay compared to 6% of children at 300% and higher of the poverty line [6]. Moreover, children from low-income families are more likely to present a more severe form of disease as measured by the number of affected teeth per child; for example, children from more affluent families had a dft (decayed, filled teeth) of 0.31, while children from poor families had a dft of 1.49 [6]. At the local level, similar findings were reported in California [8] and Arizona [9].

National data also show that racial/ethnic minority children are more likely to present dental decay than non-minority children. While 18% of white children 2–5 years of age have had caries experience, that percentage increases to 40% among Mexican-American children and 29% among black children [5]. Minority children also tended to present a more severe case of disease; the dft found in NHANES III was 0.67 for non-Hispanic whites, 1.04 for non-Hispanic blacks and 1.71 for Mexican-Americans. The difference in caries prevalence among the racial/ethnic groups was largest among children who have 6 or more decayed surfaces. Sixteen percent of all Mexican-American children, 9% of black children, and 4% of white children had 6 or more decayed surfaces compared to 13%, 10%, and 6% of children from the same racial/ethnic groups who had one or two decayed surfaces [5].

Other regional studies have also shown disparity by race/ethnicity. In California, Asian and Latino children enrolled in Head Start were more likely to have had caries experience than those from non-Hispanic white origin [8]. The highest prevalence rate for untreated ECC ever documented in the U.S. was found in Native American children living in Whitewater, Arizona. A full 95% of the 4-year-olds had untreated caries. [10].

The consistent relationship between vulnerable groups of children and prevalence of caries suggests that there are complex system-level factors that work against families in obtaining caries prevention and quality and timely oral health care.

### Trends

From the mid 1960s to 1990s there was remarkable reduction in the prevalence in dental caries in the United States for children 2 to 18 years of age [11,2]. Despite this positive trend, 2- to 5-year-old children living in poverty did not show any improvement in the prevalence of dental caries during this time period [11,12].

Not surprisingly, there is not a statistically significant difference in caries prevalence for children 2–5 years of age (objective 21.1a) between NHANES III (1988–94) and the 1999–2000 NHANES [7]. It also appears that progress in reducing caries prevalence among minority children has slowed. A report on caries prevalence among a Native American preschool population found that there were no changes between 1978–79 and 1993 [10].

Not only is the percentage of preschool children affected by decay increasing, but the severity of the caries problem also appears to be increasing. For example, among Connecticut Head Start children, the disease was more severe in 1999 than in 1991 (dmft 3.06 and 2.75 respectively) [13].

The uneven reduction in caries prevalence among preschool children by socioeconomic status and among minority children suggests that we can anticipate a widening in the gap in caries between the children at the ends of the social continuum. Furthermore, considering that previous caries is the best predictor of future caries [14] and that oral health status in childhood will influence oral health in adulthood [15], it is very likely that the current disparities in oral health among preschoolers by social characteristics will be carried through childhood and adulthood.

### Issues in obtaining dental care for ECC

The treatment of ECC requires special considerations given the pre-cooperative nature of the affected child. In most cases, dental treatment has to be provided under general anesthesia or sedation. Usually only pediatric dentists have the expertise necessary for these procedures. The result is high treatment costs. Restorative dental treatment under general anesthesia may cost anywhere between $1500 and $6000 [16-19].

Because of these high costs, dental insurance has been reported as a strong determinant of dental care utilization [2]. Children may be covered by private dental insurance or public dental insurance, *i.e*. Medicaid. While some special groups of poor children, including homeless children and undocumented, foreign-born children do not qualify for Medicaid, the vast majority of children from low-income families have dental insurance through Medicaid. Since 1998, children who are above the poverty cutoff point for Medicaid, but below state-specific thresholds, are covered by the State Children's Health Insurance Program (SCHIP).

Children enrolled in either Medicaid or SCHIP are entitled to receive dental care as part of the mandatory "Early and Periodic Screening, Diagnosis, and Treatment" (EPSDT) program. The EPSDT is the comprehensive and preventive health program for Medicaid enrollees younger than 21 years of age [2]. The oral care covered by EPSDT is far-reaching in scope and was developed with considerable insight into the unique experience of children and the importance of early screening and treatment. For example, the program covers education for parents and children, assistance in locating a provider, and aid in transportation to appointments. The Inspector General Report, issued in 1996, was a sentinel publication in oral health care because it documented the failures of Medicaid in meeting the requirements established in the EPSDT. The report documented that only 20% of all eligible children received regular preventive oral health care, although this is a mandated part of the program [20]. The report cited problems with outreach and enrollment, inconsistent eligibility of families, and low participation rates by clinicians.

Availability of dental care providers is a key problem for children from low-income families. As noted earlier, general dentists are often unwilling or unable to see young children because of the unique needs they represent; therefore, the care of preschool children is essentially under the purview of pediatric dentists. There is a documented shortage of pediatric dentists nationwide; the care of children under 5 years of age in this country is in the hands of approximately 3,500 pediatric dentists or roughly one pediatric dentist for 5,648 children (US Census Bureau). Such a shortage creates high demand; there is certainly no economic motivation for dentists to accept Medicaid patients or patients who are considered difficult to treat.

Clearly the number and type of clinicians – dentists, hygienists, allied medical personnel, primary care physicians – who can provide care should be increased. But the second part of the problem is ensuring that they will see poor and difficult to treat children. Studies have shown that increasing Medicaid reimbursement rates for dentists increases the number of children seen by participating dentists, but it does not necessarily increase the number of providers participating in the program [21,22]. More fundamental barriers to providing care for poor children may exist.

In many cases, the lack of dental care occurs because the families do not seek dental care. In a study by Mofidi and colleagues [23], families with Medicaid insurance discussed factors that dissuaded them from making and keeping dental appointments. They named such exclusionary practices as: being treated disrespectfully by the clinic staff, discrimination, long wait times, limitation in provider choice, and difficulties with transportation to the appointments. Another problem is the low perceived need among caregivers of 2–5 year olds; data from NHANES III show that while 19% of these children had normative needs (needs that are defined by a dentist), only 9% or the parents indicated a perceived need [24].

In summary, the dental care needs of low-income young children are substantial. These needs occur in an environment in which dentists practice autonomously and are free to establish a clientele that suits their needs and proclivities. Therefore, there is a contradiction in addressing dental care for preschool children. We are trying to solve a public health problem – providing preventive care for a vulnerable population while meeting tremendous needs for timely secondary and tertiary prevention–within a private dentistry model of care delivery. While public health seeks to promote health and provide care to those who need it, private dentistry provides care to those who can afford it. Unfortunately, as described above, those who are most likely to present with ECC, and thus need dental care, are also the least likely to afford it.

### Consequences of untreated ECC

Caries is a unique childhood disease; it is the most common disease of childhood that is not self-limiting. Timely professional intervention is required. But given that untreated caries is very prevalent and significant barriers exist to obtaining treatment, an unfortunate pattern occurs. As treatment for ECC is delayed, the child's condition worsens and becomes more difficult to treat, the costs of treatment increases and the number of clinicians who can perform the more complicated procedures diminishes. We have argued elsewhere that the consequences of untreated pediatric caries are, therefore, a dangerous spiral of unmet needs: as treatment is delayed, the problem becomes more serious and more difficult to treat and access issues simply multiply [24].

The most common immediate consequence of untreated dental caries is dental pain. Even though dental pain is a serious and common problem, very limited research about the epidemiology of children's dental pain has been conducted. So far we know that 28% of kindergarteners and 3rd graders with caries experience [25] and 10% of children in Head Start have complained from dental pain [24,26]. Dental pain is usually endured for several weeks and affects children's regular activities, such as eating, sleeping, and playing [27]. While there are no data yet on school performance in the presence of untreated oral pain, this factor would only add to the constellation of burdens low-income children face in achieving academically.

Tooth extraction is a common and necessary treatment for advanced caries. Premature loss of molars is likely to result in future orthodontic problems. Therefore, children affected by ECC are likely to continue having oral health problems for which treatment is often financially out of reach for their parents. Furthermore, caries in the early years has been associated with caries in late childhood [14,28,29]. A recent report from one of the few longitudinal studies on oral health has indicated that the effect of poor oral health during the early years, in addition to family low socioeconomic status, is a predictor of poor oral health during adulthood [15]. Therefore, oral health inequalities in early years are likely to persist through the adult years.

It is important to consider not only the consequences of untreated caries and untreated pain, but the consequences of the disparity in general. Untreated ECC and untreated pain must be seen in the context of other problems that fall more heavily on minority and poor children. For example, academic achievement in young children is a hallmark of intellectual, social, and emotional development. It also presages academic achievement in later years. However, it is another source of acute disparity between poor and minority children and their more privileged counterparts. Untreated caries and untreated pain constitutes another hurdle, another barrier that these children face in achieving parity in many aspects of daily living.

### Efforts to improve dental care utilization

So far, few programs to increase dental care utilization by preschool children have shown success and sustainability. Given that the dentist shortage is recognized as a serious problem, most of the successful programs target the availability of providers.

An example of such a program is the ABCD program in Washington State [30]. In this program general dentists are specially trained to see young patients. The program includes increasing the Medicaid reimbursement rates as well as participants' benefits and, importantly, there is strong outreach to enroll and engage eligible families. One of the multiple evaluations of this program indicated that, compared to children not in the ABCD program, ABCD children were more likely to have had a dental appointment, reported fewer fears, and their parents were more satisfied with their children's dental care [31].

Another successful effort to increase availability of dental care providers in underserved areas, mainly rural areas, is the Pediatric Dental Fellowship program at the University of Maryland. Pediatric dentists who have graduated from U.S. dental schools are recruited to work in community health clinics to see Medicaid patients. Fellows generally spend two years in the program and see it as a stepping stone in their careers, perhaps giving them time to prepare for their specialty board or facilitating obtaining a Maryland dental license. This program started in 1998 and currently includes 8 fellows (N. Tinanoff, personal communication).

Different versions of loan repayment programs at the federal and state levels also increase the number of dentists accepting children covered by Medicaid. Dentists in these programs are required to see a percentage of children covered by Medicaid as part of their patient load or are required to serve full time for a number of years in specified sites located in underserved areas (see: ).

Another successful program providing timely dental services to preschool children is North Carolina's "Into the Mouths of Babes." This program is unique in that it was the first to train medical providers to offer dental services such as risk assessment, screening, referral, fluoride varnish application, and caregivers' counselling. Evaluations from this program indicated non-dental professionals were able to provide preventive dental services that dentists were not providing. Children were receiving services that otherwise would not be available to them [32].

Another key policy opportunity is addressing the regulation of who can provide dental services. State Dental Licensing Boards currently control what services dental professionals can provide and the type of supervision needed. Allowing dental hygienists more independent practice in public health sites and clinics would help bridge the gap by providing early general and oral health promotion activities for infants. Expansion of functions of auxiliary dental personnel is also important to increase services for preschool children.

## Conclusion

"We have the knowledge, skills and tools to eliminate the suffering of dental disease for most children, yet somehow we have failed to put the pieces together for our most vulnerable children. Children from families with low incomes, minority children, and those with special health needs have been left behind." [33] (CE Fox, quoted in Spisak *et al*. 1998).

The best intentions, the most innovative technologies will not improve the oral health of America's children until there is a system that actually provides consistent oral health care. The fact that low-income children 2–5 years of age have not experienced a reduction in caries prevalence in the past 10 years clearly indicates that the interventions to prevent and treat ECC have not adequately reached all members of the community. We assume that the problem in oral health practices reaching the community is one of dissemination. There has been sufficient research to identify the best practices to prevent and treat caries in ideal circumstances. New research studies need to take into account the real-life circumstances of low-income groups including their experiences interacting with the dental care system.

## Competing interests

The author(s) declare that they have no competing interests.

## Authors' contributions

All authors read and approved the final manuscript.
